# Hormonal differences in perpetrators of intimate partner violence

**DOI:** 10.3389/fpsyt.2024.1432864

**Published:** 2024-07-09

**Authors:** Arthur L. Cantos, Gabriela Ontiveros, Robert K. Dearth, K. Daniel O’Leary

**Affiliations:** ^1^ Department of Psychological Science, The University of Texas Rio Grande Valley, Edinburg, TX, United States; ^2^ Department of Biology, The University of Texas Rio Grande Valley, Edinburg, TX, United States; ^3^ Department of Psychology, Stony Brook University, Stony Brook, NY, United States

**Keywords:** partner violence, testosterone, cortisol, risk factors, physical perpetration

## Abstract

**Objective:**

In order to gain a better understanding of the individual and joint impact of testosterone and cortisol on behavior, the present study was developed to test the differences in each hormone alone and conjointly between perpetrators of IPV and non-violent controls.

**Method:**

Perpetrators of IPV on probation were compared to a control group of non-aggressive males from Hidalgo County in the Rio Grande Valley on baseline testosterone and cortisol, as well as several relevant questionnaires measuring aggression and trait anger. Differences in cortisol following exposure to a stressful event were also examined. Procedures included two laboratory visits consisting of questionnaires, a number of salivary testosterone and cortisol collections, and exposure to a stressor.

**Results:**

Perpetrators had higher basal testosterone and post stressor cortisol levels than non- violent controls as well as a higher T/C ratio. In addition, trait anger moderated the relationship between both testosterone alone, and the testosterone/cortisol ratio and perpetration of IPV.

**Conclusion:**

Results are consistent with the hypothesis that testosterone leads to antisocial behavior, including perpetration of violence. The results are also consistent with the dual hormone hypothesis, i.e., that testosterone and cortisol work together to jointly regulate social dominance and aggression. Both the increased freestanding testosterone and the increased cortisol following exposure to stress places these men at risk for perpetrating violence. Clinical implications are discussed.

## Hormonal differences in perpetrators of intimate partner violence

Intimate partner violence (IPV), including physical and psychological aggression, is associated with numerous negative outcomes (e.g., depression, poor physical health) that significantly impair the functioning of individuals and their families and have high costs for society at large. For both men and women, physical IPV victimization was associated with increased risk of current poor health, depressive symptoms, substance use, developing a chronic disease, chronic mental illness, and injury (1). In a related population-based study with 4,060 women, Hispanic women in the United States who were IPV victims had disproportionate rates of depression, post-traumatic stress disorder, substance misuse, and anxiety, in decreasing orders of prevalence, i.e., with depression having the highest prevalence (2).

Despite the research on psychological characteristics of perpetrators of IPV, research into the biological factors that underlie the perpetration of IPV has been limited. With the exception of brain imaging for highly aggressive men (3), the field of aggression research has largely relied on self-report, observer-report, observation of dyads, and interview data in order to identify risk factors (e.g., personality traits, attitudes towards aggression, and exposure to aggression in the family or origin) (3, 4). Recently, organizations such as the National Institute of Mental Health have emphasized the importance of understanding phenomena through direct observable behavior as well as through neurobiological measures (5). Thus, biomarkers, or biological states that differentiate between aggressive and non-aggressive individuals, represent an important and necessary next step in the evolution of aggression research.

The field has reasonably complex multivariate psychosocial models of intimate partner aggression (6), but Raine (7) has argued for evaluations of both psychological as well as biological predictors of aggression. Accordingly, we turn to discussion of biological markers of aggression, along with analyses of how such markers might be used in an interactive or additive manner with psychosocial risk factors. Further, if one can use biological markers of aggression that can be obtained readily in an office, such as salivary hormonal assays, the practical utility of such markers increases. A literature review of biological markers of IPV perpetration (8) suggests that biological variables in the domains of head injury, neuropsychology, psychophysiology, neurochemistry, metabolism and endocrinology, and genetics play a significant role in the etiology of IPV. The authors concluded that at the most basic level, neurochemical alterations in perpetrators, specifically excessive testosterone or reduced serotonin activity, reflect an alteration of neuronal function that can be simplistically thought of as promoting rapid responding to threatening external stimuli.

An analysis of archival data from 4,462 U.S. military veterans concluded that testosterone leads directly to antisocial behavior since testosterone was correlated with a variety of antisocial behaviors for all individuals (9). However, a review of over 42 correlational studies concluded that there is a small association (*r* = 0.08) between testosterone levels and measures of aggression and that these associations were strongest for young men and offenders (10). Archer (11) also concluded that people with higher existing levels of testosterone are more likely to show higher scores on a variety of different assessments of dominance, although this is a weak relationship. The relatively weak association between testosterone and aggression has led some to argue that this weak association may be due to the failure to account for levels of a second hormone, namely, cortisol. Thus, the Dual-Hormone Hypothesis proposes that the hormones testosterone and cortisol jointly regulate social dominance and aggression in humans. The neuroendocrine systems that produce testosterone and cortisol are thought to be diametrically opposed, with cortisol modulating the effects of testosterone on aggressive psychopathology (12, 13). The combination of high levels of testosterone (associated with dominance-seeking behavior), and low levels of cortisol (associated with avoidance behavior) may be associated with increased dominance and aggression more consistently than the levels of testosterone or cortisol, individually (13).

A few studies have investigated the role of testosterone and cortisol on intimate partner violence. Romero-Martinez et al. (14) compared participants who had previously been jailed for IPV and controls matched for SES and absence of partner aggression on testosterone and cortisol levels. Their methodology involved having subjects stressed by performing the Trier Social Stress Test (TSST). IPV perpetrators experienced decreases in salivary testosterone (T) levels, a moderate worsening of mood, slight anxiety, and a salivary cortisol (C) level increase. Moreover, high basal T was related to high levels of anger, anxiety, and worse mood. Controls experienced smaller changes in T and larger changes in C and psychological mood. The authors concluded that together with social aspects involved in IPV, differences in psychobiological variables and their relationships could play a relevant role in the onset and perpetuation of violent behavior. In a follow-up study by the same lab group, Romero-Martinez et al. (15) compared IPV perpetrators with men matched for SES and no IPV. They found that while IPV perpetrators had higher antisocial, borderline, and narcissistic personality traits and anger expression than controls, they did not differ in basal T/C ratio. However, only in IPV perpetrators was there a positive relationship between these variables, the T/C ratio playing a moderating role in the relationship of antisocial and borderline traits with anger expression. This led the researchers to conclude that in IPV perpetrators the T/C ratio may explain why certain personality traits are associated with high risk of becoming violent. In a third study from the same lab group in Valencia, Spain, IPV perpetrators were compared with men with no IPV who were matched for age and SES (16).

Both the IPV perpetrators and the non IPV perpetrators were stressed using the Trier Social Stress Test. Perpetrators of IPV against women had lower salivary cortisol and higher salivary testosterone/cortisol ratio levels during the post- acute cognitive laboratory stressor period, as well as higher total levels (average) of salivary oxytocin than controls. In addition, high levels of baseline anxiety and negative affect were related to high rises in cortisol during the stress task only in the perpetrators.

An additional study from a different lab group evaluated the association of T/C with IPV of male undergraduate college students using the Trier Social Stress Task (17). Trait aggression moderated the relationship between the ratio of testosterone to cortisol (T/C) and IPV perpetration. High T/C ratio, or more testosterone relative to cortisol, was associated with elevated IPV in men low in trait aggression, whereas the association between T/C ratio and IPV was non-significant in men high in trait aggression (17). This result seems counter to the overall result of others -where Archer (10) found the association greatest in young men and criminals.

Overall, a major review by Archer et al. (10) concluded that the correlation between T and aggression is small (-.08). In addition, the association, if detected, appeared to be evident in young men and criminal offenders. Later research looked at the simultaneous role of T and C as it was believed that C would moderate or dampen the role of T. Research on the simultaneous role of T and C has proven to be complex as it is unclear if differences in T would be evident as a baseline measure in men or whether men need to be stressed to detect differences in T as some believe that rises when males are threatened. Similarly, should differences in the stress hormone, cortisol, across groups of aggressive and non-aggressive men be evident during a baseline non- stress period or only when the men are stressed, and cortisol has risen. Recent research in the T/IPV arena has used men who have been charged with physical assault against their female partners to obtain men who engage in more severe levels of aggression, T and C are evaluated simultaneously, and some stressor is used to potentially elevate both T and C.

## The present study

In order to gain a better understanding of the individual and joint impact of testosterone and cortisol on behavior, the present study was developed to test the differences in each hormone alone and conjointly between perpetrators of IPV on probation and non-violent controls.

Differences in cortisol following exposure to a stressful event were also examined. In addition, this study assessed the moderation of the testosterone hypothesis and the dual hormone hypothesis by trait anger, using measures of testosterone and cortisol for the prediction of IPV.

Procedures included two laboratory visits consisting of questionnaires, and a number of salivary testosterone and cortisol collections. First, we hypothesized that perpetrators of IPV would have higher baseline testosterone, lower cortisol than the non-aggressive controls and that perpetrators would have a greater increase from pre to post stressor cortisol. Second, we hypothesized that a ratio of high testosterone to cortisol (T/C) would differentiate between male perpetrators of IPV and those men in the control group who have no history of aggression toward their partner. A third prediction was that trait anger would moderate the relationship between testosterone and perpetration of IPV. In addition, the current study proposed to test the Hypothesis 4) moderation of the dual hormone hypothesis by trait anger, using measures of testosterone and cortisol for the prediction of IPV.

## Method

### Sample

The sample was composed of 60 adult male volunteers: 30 perpetrators of IPV and 30 men without a history of IPV perpetration. The IPV volunteers were recruited from the Hidalgo

County Probation Department in Edinburg, Texas in the Rio Grande Valley (RGV). Adults who were on probation for IPV were asked to participate voluntarily in this study. The inclusion criteria were as follows: (a) the participant is a male, (b) is at least 18 years of age, (c) on probation for an IPV-related offense in the RGV and (d) not currently taking medication which would interfere hormone measurements. The control sample of men who had no history of IPV were recruited via flyers distributed at local community centers.

## Measures

### Demographics and socioeconomic status

Demographic characteristics were assessed using single items, and they included age, race/ethnicity, highest level of education, and annual salary. The highest level of education was assessed using five categories: (1) *less than 4^th^ grade*; (2) *high school diploma*; (3) *associate degree*; (4) *bachelor’s degree*; and (5) *master’s degree*. Similarly, annual salary was assessed using five categories (1) *less than $10,000*; (2) *$11,000-$20,000*; (3) *$21,000-$30,000*; (4) *$31,000-$45,000*; and (5) *$45,000 or more*.

### Physical intimate partner violence perpetration

Physical IPV perpetration was measured using the physical assault subscale of the Revised Conflict Tactics Scale-2, the most widely used measure in the field of IPV (CTS-2) (18). The CTS-2, a 39-item scale (78 questions), is used to assess instances of five types of abusive behavior within the last twelve months: Negotiation, Psychological Aggression, Physical Assault, Sexual Coercion, and Injury. Questions are paired; respondents first answer regarding their behavior towards a partner in a dating, cohabiting, or marital relationship and then their partner’s behavior towards them. Items are rated on a seven-point Likert scale system with the following distinctions: 1 = *Once in the past year*, 2 = *Twice in the past year*, 3 = *3-5 times in the past year*, 4 = *6-10 times in the past year*, 5 = *11-20 times in the past year*, 6 = *More than 20 times in the past year*, 7 = *Not in the past year, but it did happen before*, 0 = *This has never happened*. This scale demonstrates sound psychometric properties, with mean internal consistency of the CTS-2 to be.77. To analyze physical assault in the present study, the 12 items that constitute the physical assault scale were analyzed as outlined by Straus et al. (18). A variable was created that comprised the sum of all 12 items that load into the physical assault scale with higher scores indicating a higher frequency of physical IPV. Internal consistency for the present study was α = .51 for the physical assault scale.

### State trait anger expression

The State-Trait Anger Expression Inventory 2 (STAXI-2) (19) measures the intensity of anger as an emotional state (State Anger) and the disposition to experience angry feelings as a personality trait (Trait Anger). It consists of 57 items that load into 6 scales and an Anger Expression Index (total anger expression score). This scale is rated on a 4-point Likert scale system that assesses intensity of anger at a particular moment and the frequency of anger experience, expression, and control. This well-known anger measure has supported data for high reliability and validity. Alpha coefficients for the normative data, including both the general and psychiatric population, were above.84 for all scales and subscales, except for Trait Anger/Angry Reaction (assesses the respondent’s angry reaction to negative situations) which had an alpha coefficient of.76 and.73 for women and men, respectively. Based on normative data factor analyses and factor loadings, support is available for the construct validity of the STAXI-II.

The present study used the sum score of the 15 items that load into the state anger subscale (internal consistency: α = 0.73) and the 10 items that load into the trait anger subscale(internal consistency: α = 0.84). A higher score was indicative of higher anger intensity as an emotional state and higher personality dimension of anger proneness.

### Testosterone-cortisol ratio

Saliva samples were measured using a human testosterone and cortisol ELISA from Enzo Life Sciences (Farmingdale, NY, USA: Cat# ADI-900-176 and ADI-900-071). The assay sensitivity was 56.72 and 2.6 pg/mL (picograms per milliliter) for cortisol and testosterone, respectively. Samples were measured in duplicate, and the mean sample was utilized in our analyses. The curve was a standard curve using known concentrations included in the kit of the respective hormones. Good precision was obtained, with inter-assay and intra-assay variation coefficients for cortisol and testosterone of less than 10%. The concentration of cortisol and testosterone was expressed as pg/mL.

## Procedure

Participants were individually briefed about the research plan, received, and signed the informed consent form, with all the required provisions explained. Participants were seen in the morning on two occasions to complete the research questionnaires via an unstructured interview format. Research participants were interviewed at a designated office at the Hidalgo County Probation Department and control participants were interviewed at a research office located at the university psychology training clinic. All study protocols were reviewed and approved by the Institutional Review Board of the University of Texas Rio Grande Valley in accordance with the declaration of Helsinski.

*Session 1.* It was requested that participants avoid eating a major meal, foods with high sugar or acidity, high caffeine content, alcohol, nicotine, or drugs (prescription/over-the-counter- medication), brushing their teeth, or doing exercise two hours before arriving to their appointment. Participants were then asked to provide two saliva samples for hormonal analysis: Drool 1 was gathered at the beginning of the session and Drool 2 was gathered at the end of the session. Questionnaires administered during this session included a sociodemographic questionnaire and the CTS-2 (18). Participants were asked what time they woke up the morning of session 1 and this time was recorded and used as a control variable in analyses to control the natural diurnal cycle of cortisol. The sampling of saliva was non-invasive; the participant was asked to slowly drool into a straw which was attached to a small plastic vial. Research assistants immediately secured the vial and placed it in a -20-degree Celsius freezer to be transferred to the university endocrinology research laboratory.

*Session 2.* Participants were asked what time they woke up and this time was again recorded. A third saliva sample (Drool 3) was obtained. Participants then proceeded to complete the State Trait Anger Expression Inventory-2 (STAXI-2) (19). The participants then engaged in a stress induction exercise that differed for control and research participants. Control participants were asked to speak and describe about the most stressful situation they had experienced in the last 12 months, and research participants were asked to speak about the situation that led them to be placed on probation and asked to describe if they believed it was fair that they were arrested and mandated to probation. Subsequently, Drool 4 was collected, and the STAXI-2 was administered again. After 20 minutes, Drool 5 was obtained followed by a third administration of the STAXI-2, and 20 minutes later, Drool 6 was obtained followed by the fourth administration of the STAXI-2.

## Results

### Descriptive statistics

The total sample of mostly Hispanic men (98.3%) consisted of 30 control participants from the community and 30 men placed on probation for perpetrating intimate partner violence. Demographic characteristics of the study sample are provided in **Table 1.**

**Table 1:** Demographic characteristics of the total sample (*N* = 60).

	Variable	N (%)
Age		27.47 (7.4)^a^
	18-23	22 (36.7)
	24-29	19 (31.7)
	30-35	10 (16.7)
	36-41	6 (10.0)
	42-47	2 (3.3)
	48-51	1 (1.7)
Race/Ethnicity		
Hispanic/Latino		59 (98.3)
African American		1 (1.7)
Highest Level of Education		12.52 (2.90)^a^
Less than 4^th^ grade		4 (6.7)
High School Diploma		33 (55.0)
Associate degree		9 (15.0)
Bachelor’s degree		14 (23.3)
Annual Salary		18.70 (11.54)^b^
Less than $10,000		28 (46.7)
$11,000-$20,000		7 (11.7)
$21,000-$30,000		8 (13.3)
$31,000-$45,000		12 (20.0)
$45,000 or more		5 (8.3)

^a^Mean (SD) provided; ^b^Mean (SD); Gross yearly family income in thousands of dollars.

**Table 2** provides group means, standard deviations, percentages, and comparisons between the control and research group participants on demographic variables. Independent samples t-tests were conducted to examine differences in age, education, and income between groups. The results of these tests indicated that there was a significant difference in age observed between the control group (*M* = 24.13, *SD* = 4.28) and the research group (*M*= 30.80, *SD* = 8.43), *t*(58) = -3.86, *p* <.001. There was also a significant difference in education observed between the control group (*M* = 13.53, *SD* = 2.61) and the research group (*M*= 11.50, *SD* = 2.86), *t*(58) = 2.88, *p* <.05.

**Table 2:** Differences in demographic variables in control and research participants (*N* = 60).

Variable	Control (*n* = 30)		Research (*n* = 30)	
	M	SD	M	SD
Age (years)*	24.13	4.28	30.80	8.43
Education (years)*	13.53	2.61	11.50	2.86
Race/Ethnicity				
Hispanic/Latino	30		29	
African American	0		1	

*statistically significant difference.

## Hypothesis 1a and 1b

There was a significant difference in testosterone in session 1 for the control group (*M* = 334.00, *SD* = 110.73) and the research group (*M*= 413.89, *SD* = 126.11), *t*(58) = -2.61, *p* <.05, *d*= 0.66. There was also a significant difference in testosterone in session 2 for the control group (*M* = 325.81, *SD* = 103.52) and the research group (*M*= 417.48, *SD* = 128.27), *t*(58) = -3.05, *p* <.05, *d* = 0.78. There were no significant differences between the control and research group in cortisol in session 1 (*M*= 6513.33, *SD* = 549.50 and *M* = 6535.00, *SD* = 412.72, respectively), session 2 (*M*= 6647.67, *SD* =449.31 and *M* = 6680.67, *SD* = 476.84, respectively) and before the stressor (*M*= 6667.67, *SD* =429.38 and *M* = 6682.33, *SD* = 459.78, respectively). A significant difference in cortisol after the stressor was found between the control group (*M* = 6681.67, *SD* = 586.30) and the research group (*M*= 7219.33, *SD* = 435.07), *t*(58) = -4.03, *p* <.001, *d* = 1.04. **Table 3**

**Table 3:** Differences in testosterone and cortisol in control and research participants (*N* = 60).

Hormones	Control (*n* = 30)		Research (*n* = 30)	
	M	SD	M	SD
Testosterone Session 1*	334	110.73	413.89	126.11
Testosterone Session 2*	325.81	103.52	417.48	128.27
Cortisol Session 1 Sample	6513.33	549.5	6535	412.72
Cortisol Session 2 Sample	6647.67	449.31	6680.67	476.84
Cortisol Before Stressor	6667.67	429.38	6682.33	459.78
Cortisol After Stressor*	6681.67	586.3	7219.33	435.07

*statistically significant difference

## Hypothesis 2

The second hypothesis was that a ratio of high testosterone to cortisol (T/C) would differentiate men placed on probation for IPV and men in the control group with no history of aggression. An independent samples t-test was conducted to compare the T/C ratio levels in the control and research groups in session 1 and session 2. There was a significant difference in the T/C ratio in session 1 for the control group (*M* = .05, *SD* = .02) and the research group (*M*= .06, *SD* = .02), *t*(58) = -2.55, *p* <.05, *d* = 0.66. In addition, a significant difference in the T/C ratio was found in session 2 for the control group (*M* = .05, *SD* = .01) and the research group (*M*= .06, *SD* = .02), *t*(58) = -3.00, *p* <.05, *d* = 0.77. **Table 4**

**Table 4:** Differences in T/C ratio in session 1 and 2 (*N* = 60).

Ratio	Control (*n* = 30)		Research (*n* = 30)	
	M	SD	M	SD
Testosterone Day 1 / Cortisol Day 1*	.05	.02	.06	.02
Testosterone Day 2 / Cortisol Day 2*	.05	.01	.06	.02

*statistically significant difference.

## Hypothesis 3

The third hypothesis predicted that the levels of testosterone would be positively related to perpetration of IPV as reflected on scores of the Physical Assault scale of the CTS-2 scale.

There was a positive correlation between the Physical Assault scale and the testosterone value of session 2, *r* (60) = .26, *p* <.05. In addition, when the mean of the two testosterone values (session 1 and session 2) was obtained, it was also positively related to perpetration of IPV, *r* (60) = .25, *p* <.05.

## Hypothesis 4

The fourth hypothesis predicted that trait anger would moderate the relationship between testosterone at both session 1 and 2 and perpetration of IPV. Given that age was significantly associated with testosterone at session 1 and 2 (*r*(60)=-44, *p* = .001; *r*(60)=-41, *p* = .001, respectively), it was included in the models as a control variable. Results from the binary logistic regression assessing the relationship between trait anger, testosterone at session 1 and physical IPV perpetration indicated that testosterone at session 1 (B = .08, *p* = .01), and trait anger (B = 1.41, *p* = .01) were significant predictors of physical IPV perpetration. When the moderation of trait anger on the association between testosterone at session 1 and physical IPV perpetration was assessed, results indicated that there was a significant interaction (B = -.003, *p*= .03), thus there was a moderation effect of trait anger on the relationship between testosterone at session 1 and IPV perpetration.

When the relationship between trait anger, testosterone at session 2 and physical IPV perpetration was assessed via binary logistic regression, testosterone at session 2 (B = .09, *p* =.01), and trait anger (B = 1.61, *p* = .01) were significant predictors of physical IPV perpetration.

In addition, the moderation of trait anger on testosterone at session 2 and IPV perpetration was also significant (B = -.004, *p* = .02). **Table 5**

**Table 5:** Binary logistic regression models: trait anger as a moderator of the relationship between testosterone at session 1 and 2 and physical IPV perpetration.

Variables	Nagelkerke R^2^	B (OR)	95% CI for OR
Physical IPV Perpetration			
	.81		
Age		.64 (1.89)**	[1.32,2.79]
Testosterone Session 1		.08 (1.08)*	[1.17,2.47]
Trait Anger		1.41 (4.08)*	[1.38,13.90]
Trait Anger x Testosterone Session 1		-.003 (1.00)*	[.96, 1.00]
Physical IPV Perpetration			
	.83		
Age		.64 (1.91)*	[1.28,2.85]
Testosterone Session 2		.09 (1.10)*	[1.03,1.17]
Trait Anger		1.61 (5.01)*	[1.44, 17.44]
Trait Anger x Testosterone Session 2		-.004 (1.00)*	[0.99, 1.00]

^*p < 0.05. **p < 0.01. Values were rounded to the nearest tenth. OR, odds ratio. CI, confidence interval. Lines in between indicate separate regression models. Dependent variables are in bold.^

## Hypothesis 5

The current study proposed to test the moderation of the dual hormone hypothesis by trait anger, using measures of testosterone and cortisol for the prediction of IPV. Results from the binary logistic regression assessing the relationship between trait anger, the T/C ratio at session 1 and physical IPV perpetration indicated that the T/C ratio at session 1 and trait anger were significant predictors of physical IPV perpetration (B = 530.68, *p* <.01; B = 1.48, *p* = .01, respectively). When the moderation of trait anger on the association between the T/C ratio at session 1 and physical IPV perpetration was assessed, results indicated that there was a significant interaction (B = -.02, *p* = .03), thus there was a moderation effect of trait anger on the relationship between the T/C ratio at session 1 and IPV perpetration.

When the relationship between trait anger, the T/C ratio at session 2 and physical IPV perpetration was assessed via binary logistic regression, the T/C ratio at session 2, and trait anger were significant predictors of physical IPV perpetration (B = 930.00, *p* <.01; B = 2.39, *p* = .01, respectively). In addition, the moderation of trait anger on the T/C ratio at session 2 and IPV perpetration was significant (B = -42.57, *p* = .02). **Table 6**

**Table 6:** Binary logistic regression models: Trait anger as a moderator of the relationship between T/C ratio at session 1 and 2 and physical IPV perpetration.

Variables	Nagelkerke R^2^	B (OR)	95% CI for OR
Physical IPV Perpetration			
	.82		
Age		.65 (1.92)**	[1.32,2.79]
T/C Ratio Session 1		530.68 (2.95E+23)*	[2.98E+67,-]
Trait Anger		1.48 (4.38)*	[1.38,13.90]
Trait Anger x T/C Ratio Session 1		-22.48 (.00)*	[.00,.09]
Physical IPV Perpetration			
	.88		
Age		.80 (2.22)**	[1.30,3.77]
T/C Ratio Session 2		930.00 (-)**	[7.39E+103,-]
Trait Anger		2.39 (10.94)*	[1.65, 72.33]
Trait Anger x T/C Ratio Session 2		-42.57 (00)*	[.00,.00]

^*p < 0.05. **p < 0.01. Values were rounded to the nearest tenth. OR, odds ratio. CI, confidence interval. Lines in between indicate separate regression models. Dependent variables are in bold.^

## Discussion

As predicted, perpetrators of IPV had higher baseline testosterone than the non- aggressive controls on both days, despite the controls being significantly younger. In addition, as predicted, levels of testosterone were found to be positively related to perpetration of IPV as reflected on scores on the CTS-2. Contrary to prediction, perpetrators did not differ from the controls on baseline cortisol but as predicted, the perpetrators showed a larger increase in cortisol than the controls following exposure to a stressor, probably reflecting the fact that speaking about the stress of being arrested for IPV was a significant stressor for the perpetrators. We believe that the use of a stressor such as the report of being arrested for IPV is more ecologically valid than the use of the often-used Trier stressor which involves solving difficult math problems.

These results are consistent with the hypothesis that testosterone leads to antisocial behavior, including perpetration of violence since perpetrators had significantly higher levels of testosterone than non-aggressive controls and testosterone was correlated with measure of perpetration of intimate partner violence. These results are consistent with results of a study with a large military sample of over 4,462 military veterans, in which testosterone was associated with men’s physical aggression to their wives (20) and a study which found elevated testosterone levels were associated with both verbal and physical aggression toward an intimate partner in culturally diverse men of low socioeconomic status who had a main sexual partner (r=.24) (21).The results are further consistent with a review of over 42 correlational studies which concluded that there is a small association (*r* = 0.08) between testosterone levels and measures of aggression which were strongest for young men and offenders (10). The finding that perpetrators were higher than controls in freestanding levels of testosterone is consistent with the first hypothesis that the testosterone response to challenge increases aggression since perpetrators are starting off with higher levels of testosterone even before being exposed to the challenge. A single administration of testosterone has been shown to rapidly modulate men’s perceptions of their own physical dominance, which may possibly explain the link between testosterone and dominance related behaviors (22). Testosterone has also been shown to causally modulate emotional decision making and to increase affective sensitivity (23).

There was no difference on baseline cortisol between perpetrators and controls. This finding is somewhat inconsistent with the literature. However, in a review of the literature on the relationship between cortisol and aggression, Salis (17) suggested that although the wealth of the evidence indicates hypocortisolism is related to aggressive behavior, a number of studies found no association and or that the relationship may be reversed depending on the characteristics of the sample and that the relationship between cortisol and aggression may depend on a number of different factors. Studies have shown that certain characteristics in conjunction with aggressive behavior may also lend themselves towards higher rather than low cortisol (17, 24). Cima, Smeets and Jelicic (25) found that non psychopathic aggressive men had high diurnal cortisol. Given that the perpetrators in the present study were found to engage mostly in reactive violence (26), it may be that the sample of perpetrators in this study were mostly non psychopathic as evidenced by the higher cortisol levels. In addition, previous studies have shown that type II or reactive perpetrators present a hyper-reactivity in anticipation of stress (27), so it could be that, in addition, the fact that they were interviewed at the department of probation, the stress associated with reminder of their being on probation produced a stress response and associated rise in cortisol.

As predicted, perpetrators of IPV evidenced a greater increase in cortisol following exposure to a naturalistic stressor, than non-aggressive controls, indicating that perpetrators of IPV are more reactive to stress. Cortisol has been shown to increase after exposure to stress (28). The results of this study suggest that in addition to the higher risk involved due to the higher testosterone, their greater response and reactivity to stress, as indicated by the increase in cortisol, places them at even higher risk for perpetrating intimate partner violence in situations where their coping resources are taxed.

As predicted, a ratio of high testosterone to cortisol (T/C) differentiated men placed on probation for IPV and men in the control group with no history of partner aggression. On both days, this ratio was higher for perpetrators than the non-violent controls. These results are consistent with results from studies that reported that consistent with the “Dual-Hormone Hypothesis,” which proposes that in humans, the hormones testosterone and cortisol work together to jointly regulate social dominance and aggression, greater T/C ratios were associated with greater aggression (29, 30). However, other studies have shown that testosterone was positively related to aggression/violent crime only among low-cortisol individuals but not among high-cortisol individuals (31, 32).

## Summary of findings

Perpetrators had higher testosterone and post stressor cortisol levels than non-violent controls as well as a higher T/C ratio. In addition, trait anger moderated the relationship between both testosterone alone, and the testosterone/cortisol ratio and perpetration of IPV, indicating that the hormonal effect is more pronounced in perpetrators of IPV that have higher levels of trait anger. Subregions of prefrontal cortex, insula, amygdala, basal ganglia and hippocampus play a major role within neural networks related to aggression and have been consistently implicated in biology of aggression (33). Prototypical cases of impulsive aggression, those associated with anger, involve the recruitment of the acute threat response system structures; that is, the amygdala, hypothalamus, and periaqueductal gray (34). Results are consistent with the hypothesis that testosterone leads to antisocial behavior, including perpetration of violence and are also consistent with the dual hormone hypothesis, that testosterone and cortisol work together to jointly regulate social dominance and aggression, with greater T/C ratios being associated with greater aggression (29, 30). The increased testosterone places these men at risk for perpetrating violence as a result of testosterone’s influence on making more automatic judgements, biased by emotional factors due to a higher emotional sensitivity in conflictual situations (23). The increased cortisol following exposure to a stressor also suggests that perpetrators react more intensely to stress which further places them at risk for perpetration of violence and could explain the fact that the majority of the violence perpetrated is reactive (26).

## Clinical implications

The results of this study suggest that it would be important to assess hormonal patterns, specifically, testosterone and cortisol, in addition to personality characteristics, such as trait anger, impulsivity and psychopathy, and that this assessment might lead to fine tuning interventions designed to help reduce the level of recidivism of these perpetrators. For example, future studies might show that perpetrators high in testosterone and low in cortisol, who are supposedly the more callous, psychopathic perpetrators, who engage in intimate terrorism, would derive more benefit from an intervention based on power and control. Alternatively, perpetrators high in testosterone and higher on cortisol, might benefit from interventions addressing anger and impulse control. Irrespective of the combinations of hormonal patterns and personality characteristics of perpetrators, perpetrators high on testosterone are prone to responding aggressively, and would benefit from skills training and relapse prevention types of interventions which would train perpetrators in responding non aggressively to high-risk situations. Just providing perpetrators high on testosterone with information regarding their propensity to react to certain types of situations in an aggressive manner might have a beneficial impact with respect to curtailing their aggression (35).

It is important that pretreatment assessments for perpetrators of intimate partner violence acknowledge the heterogeneity involved in both the type of violence committed, such as reactive/proactive, self-defense, intimate terrorism, mutual combat, heterosexual, LGBT, Trans, as well as the characteristics of the perpetrators including, biological, hormonal, head injury, family only/generally violent, attachment issues, borderline personality issues, impulse control issues, anger profiles, experiential avoidance, history of trauma, alcohol and substance use, power and control issues, stage of motivation to change, underclass variables and culture identification (36). Given the heterogeneity involved, there is no one treatment that can address all the issues and it is incumbent on the providers to conduct a comprehensive assessment prior to assigning the perpetrators to a lengthy intervention which might be inappropriate to address their treatment needs, does little to nothing to reduce recidivism and simply places the victims at greater risk and only serves to misguide the public into thinking that something is being done to address the violence that is being perpetrated against significant others in the context of intimate relationships. The field needs to continue to conduct experimental studies to assess specific intervention outcomes in order to be able to address Gordon Paul’s (37) epic question: What treatment, by whom is most effective for this individual with that specific problem, and under which set of circumstances.

Research on testosterone and IPV needs to have replications across several labs. The research by Romero and colleagues (14–16) in Spain is well executed and it has been conducted with men arrested for IPV. Based on the Archer et al. (10) review, the strength of the association of T and general aggression is small (.08), and the association of T and IPV in the present study was also small (r= .28) but numerically larger than in the large review. However, the review noted that the association of T and aggression was larger with young men andoffenders. The association herein was with offenders, and we do not know what the association of T and IPV would be in a general population. It might well be less. The ratio of T/C in the research group, the partner aggressive men, was higher than in the control group, the non-aggressive men, but the ratio differences were extremely small, and ratios are notable for being less reliable than a simple mean. The role of cortisol as a stress hormone is very well established as stress increases cortisol increases. In addition, the diurnal nature of cortisol is well known with cortisol being high in the morning and low at night. However, the correlation of cortisol and aggression is unclear in humans (38) and replications are needed with clinical and representative samples to evaluate the role of cortisol, testosterone and IPV.

## Limitations

First and foremost, it needs to be understood that this is a small sample size and that the perpetrators are from a border city in Texas which is mostly, 90%, Hispanic and the results may not be generalizable to the rest of the population of men who aggress against their partner.

However, it is also a strength in that it is a homogenous population with respect to ethnicity and it contributes to an understanding of the characteristics of Hispanic (mostly of Mexican American origin) perpetrators of intimate partner violence on probation, in a specific region of the US. Second, the alpha for the physical assault scale of the CTS was low (.51). There is a concern with using the CTS, which is a self-report scale, for perpetrators to report on their use of physical violence towards their partner because the men tend to minimize the violence they perpetrated and most of their scores were near zero, in spite of the fact that they have been placed on probation following an arrest and the victim has described that they committed violence as reflected in the police reports.

Third, both groups were exposed to slightly different stressors. The assumption was that talking about being arrested would be an ecologically valid stressor for the perpetrators and that talking about the most stressful situation they experienced in the past year would be an equally ecologically valid stressor for the control group. In any case, both stressors are more ecologically valid than conducting a mathematical test, making a presentation or exposure to color words printed in different colors of ink, which are often used psychological stressors in experimental studies.

## Data availability statement

The raw data supporting the conclusions of this article will be made available by the authors, without undue reservation.

## Ethics statement

The studies involving humans were approved by University of Texas Rio Grande Valley IRB. The studies were conducted in accordance with the local legislation and institutional requirements. The participants provided their written informed consent to participate in this study.

## Author contributions

AC: Conceptualization, Formal analysis, Funding acquisition, Investigation, Methodology, Project administration, Resources, Supervision, Validation, Visualization, Writing – original draft, Writing – review & editing. GO: Formal analysis, Writing – review & editing. RD: Formal analysis, Supervision, Writing – review & editing. DO’L: Writing – review & editing.

## References

[1] Coker AL Davis KE Arias I Desai S Sanderson M Brandt HM. Physical and mental health effects of intimate partner violence for men and women. Am J Prev Med. (2002) 23:260–8. doi: 10.1016/S0749-3797(02)00514-7 12406480

[2] Reyes ME Simpson L Sullivan TP Contractor AA Weiss NH. Intimate partner violence and mental health outcomes among Hispanic women in the United States: A scoping review. Trauma Violence Abuse. (2021) 24:809–27. doi: 10.1177/15248380211043815 PMC13110112 34779327

[3] Raine A Buchsbaum M LaCasse L. Brain abnormalities in murderers indicated by positron emission tomography. Biol Psychiatry. (1997) 42:495–508. doi: 10.1016/S0006-3223(96)00362-9 9285085

[4] Stith SM Smith DB Penn CE Ward DB Tritt D. Intimate partner physical abuse perpetration and victimization risk factors: A meta-analytic review. Aggression Violent Behav. (2004) 10:65–98. doi: 10.1016/j.avb.2003.09.001

[5] Mittal VA Wakschlag LS. Research domain criteria (RDoC) grows up: Strengthening neurodevelopmental investigation within the RDoC framework. J Affect Disord. (2017) 216:30–5. doi: 10.1016/j.jad.2016.12.011 PMC5471127 28010957

[6] O'Leary KD Smith Slep AM O'Leary SG. Multivariate models of men's and women's partner aggression. J Consulting Clin Psychol. (2007) 75:752–64. doi: 10.1037/0022-006X.75.5.752 17907857

[7] Raine A. Biosocial studies of antisocial and violent behavior in children and adults: A review. J Abnormal Child Psychol. (2002) 30:311–26. doi: 10.1023/A:1015754122318 12108763

[8] Pinto LA Sullivan EL Rosenbaum A Wyngarden N Umhau JC Miller MW. Biological correlates of intimate partner violence perpetration. Aggression Violent Behav. (2010) 15:387–98. doi: 10.1016/j.avb.2010.07.001 PMC3564655 23393423

[9] Dabbs JM Morris R. Testosterone, social class, and antisocial behavior in a sample of 4,462 men. psychol Sci. (1990) 1:209–11. doi: 10.1111/j.1467-9280.1990.tb00200.x

[10] Archer J Graham-Kevan N Davies M. Testosterone and aggression: A reanalysis of Book, Starzyk, and Quinsey's (2001) study. Aggression Violent Behav. (2005) 10:241–61. doi: 10.1016/j.avb.2004.01.001

[11] Archer J. Testosterone and human aggression: an evaluation of the challenge hypothesis. Neurosci Biobehav Rev. (2006) 30:319–45. doi: 10.1016/j.neubiorev.2004.12.007 16483890

[12] Montoya ER Terburg D Bos PA van Honk J. Testosterone, cortisol, and serotonin as key regulators of social aggression: A review and theoretical perspective. Motivation Emotion. (2012) 36:65–73. doi: 10.1007/s11031-011-9264-3 22448079 PMC3294220

[13] Terburg D Morgan B van Honk J. The testosterone-cortisol ratio: A hormonal marker for proneness to social aggression. Int J Law Psychiatry. (2009) 32:216–23. doi: 10.1016/j.ijlp.2009.04.008 19446881

[14] Romero-Martínez Á. Lila M Sariñana-González P González-Bono E Moya- Albiol L. High testosterone levels and sensitivity to acute stress in perpetrators of domestic violence with low cognitive flexibility and impairments in their emotional decoding process: A preliminary study. Aggressive Behav. (2013) 39:355–69. doi: 10.1002/ab.21490 23677518

[15] Romero-Martínez Á. Lila M Moya-Albiol L. Empathy impairments in intimate partner violence perpetrators with antisocial and borderline traits: A key factor in the risk of recidivism. Violence Victims. (2016) 31:347–60. doi: 10.1891/0886-6708.VV-D-14-00149 26830110

[16] Romero-Martínez Á. Blanco-Gandía MC Rodriguez-Arias M Lila M Moya- Albiol L. Hormonal differences in intimate partner violence perpetrators when they cope with acute stress: A pilot study. Int J Environ Res Public Health. (2021) 18:5831. doi: 10.3390/ijerph18115831 34071628 PMC8198212

[17] Salis KL. *The relationship between cortisol, testosterone, and intimate partner violence: Testing the Dual-Hormone Hypothesis* (Publication No. 10188704) [Doctoral dissertation, State University of New York at Stony Brook]. ProQuest Dissertations Publishing. (2017).

[18] Straus MA Hamby SL Boney-McCoy S Sugarman DB. The revised Conflict Tactics Scales (CTS2): Development and preliminary psychometric data. J Family Issues. (1996) 17:283–316. doi: 10.1177/019251396017003001

[19] Spielberger CD. STAXI-2: State-Trait Anger Expression Inventory professional manual. Odessa, Fl: Psychological Assessment Resources (1999).

[20] Booth A Dabbs JM Jr. Testosterone and men’s marriages. Soc Forces (1993) 72:463–77doi: 10.2307/2579857

[21] Soler H Vinayak P Quadagno D. Biosocial aspects of domestic violence. Psychoneuroendocrinology. (2000) 25:721–39. doi: 10.1016/S0306-4530(00)00022-6 10938451

[22] Welling LL Moreau BJ Bird BM Hansen S Carré JM. Exogenous testosterone increases men’s perceptions of their own physical dominance. Psychoneuroendocrinology. (2016) 64:136–42. doi: 10.1016/j.psyneuen.2015.11.016 26671006

[23] Wu Y Clark L Zilioli S Eisenegger C Gillan CM Deng H. Single dose testosterone administration modulates emotional reactivity and counterfactual choice in healthy males. Psychoneuroendocrinology. (2018) 90:127–33. doi: 10.1016/j.psyneuen.2018.02.018 29482135

[24] Miller GE Chen E Zhou ES. If it goes up, must it come down? Chronic stress and the hypothalamic-pituitary-adrenocortical axis in humans. psychol Bull. (2007) 133:25–45. doi: 10.1037/0033-2909.133.1.25 17201569

[25] Cima M Smeets T Jelicic M. Self-reported trauma, cortisol levels, and aggression in psychopathic and non-psychopathic prison inmates. Biol Psychol. (2008) 78:75–86. doi: 10.1016/j.biopsycho.2007.12.011 18304719

[26] Ontiveros G Cantos A O’Leary KD. Differences among perpetrators of intimate partner violence utilizing proactive versus reactive aggression. Behav Psychology/Psicología Conductual. (2023) 31:501–23. doi: 10.51668/bp.8323300

[27] Romero-Martínez A Nunes-Costa R Lila M Gonzalez-Bono E Moya-Albiol L. Cardiovascular reactivity to a marital conflict version of the Trier social stress test in intimate partner violence perpetrators. Stress. (2014) 17:321–7. doi: 10.3109/10253890.2014.919448 24766372

[28] Cay M Ucar C Senol D Cevirgen F Ozbag D Altay Z. Effect of increase in cortisol level due to stress in healthy young individuals on dynamic and static balance scores. Northern Clinics Istanbul. (2018) 5:295–301. doi: 10.14744/nci.2017.42103 PMC6371989 30859159

[29] Manigault AW Zoccola PM Hamilton K Wymbs BT. Testosterone to cortisol ratio and aggression toward one's partner: Evidence for moderation by provocation. Psychoneuroendocrinology. (2019) 103:130–6. doi: 10.1016/j.psyneuen.2019.01.018 30682629

[30] Platje E Popma A Vermeiren RRJM Doreleijers TAH Meeus WHJ van Lier PAC. Testosterone and cortisol in relation to aggression in a non-clinical sample of boys and girls. Aggressive Behav. (2015) 41:478–87. doi: 10.1002/ab.21585 25736033

[31] Dabbs JM Jr Jurkovic GJ Frady RL. Salivary testosterone and cortisol among late adolescent male offenders. J Abnormal Child Psychol. (1991) 19:469–78. doi: 10.1007/BF00919089 1757712

[32] Popma A Doreleijers TA Jansen LM Van Goozen SH Van Engeland H Vermeiren R. The diurnal cortisol cycle in delinquent male adolescents and normal controls. Neuropsychopharmacology. (2007) 32:1622–8. doi: 10.1038/sj.npp.1301289 17228341

[33] Cupaioli FA Zucca FA Caporale C Lesch KP Passamonti L Zecca L. The neurobiology of human aggressive behavior: Neuroimaging, genetic, and neurochemical aspects. Prog In Neuropsychopharmacol And Biol Psychiatry. (2021) 106:110059. doi: 10.1016/j.pnpbp.2020.110059 32822763

[34] Blair RJ. The neurobiology of impulsive aggression. J Of Child And Adolesc Psychopharmacol. (2016) 26:4–9. doi: 10.1089/cap.2015.0088 26465707 PMC4779272

[35] Lane RD Subic-Wrana C Greenberg L Yovel I. The role of enhanced emotional awareness in promoting change across psychotherapy modalities. (2022) J Psychother Integr 32(2):131–50. doi: 10.1037/int0000244

[36] Cantos AL O'Leary KD. One size does not fit all in treatment of intimate partner violence. Partner Abuse. (2014) 5:204–36. doi: 10.1891/1946-6560.5.2.204

[37] Paul GL. Strategy of outcome research in psychotherapy. J Consulting Psychol. (1966) 31:109. doi: 10.1037/h0024436 5342732

[38] Armstrong T Wells J Boisvert DL Lewis RH Cooke EM Woeckener M. An exploratory analysis of testosterone, cortisol, and aggressive behavior type in men and women. Biol Psychol. (2021) 161:108073. doi: 10.1016/j.biopsycho.2021.108073 33727106

